# Vocal Recruitment for Joint Travel in Wild Chimpanzees

**DOI:** 10.1371/journal.pone.0076073

**Published:** 2013-09-25

**Authors:** Thibaud Gruber, Klaus Zuberbühler

**Affiliations:** 1 Centre Norbert Elias, EHESS, Marseille, France; 2 School of Psychology, University of St Andrews, St Andrews, Scotland, United Kingdom; 3 Anthropological Institute, University of Zurich, Zurich, Switzerland; 4 Cognitive Science Centre, University of Neuchatel, Neuchatel, Switzerland; 5 Budongo Conservation Field Station, Masindi, Uganda; Max Planck Institute for Evolutionary Anthropology, Germany, Germany

## Abstract

Joint travel is a common social activity of many group-living animals, which requires some degree of coordination, sometimes through communication signals. Here, we studied the use of an acoustically distinct vocalisation in chimpanzees, the ‘travel hoo’, a signal given specifically in the travel context. We were interested in how this call type was produced to coordinate travel, whether it was aimed at specific individuals and how recipients responded. We found that ‘travel hoos’ were regularly given prior to impending departures and that silent travel initiations were less successful in recruiting than vocal initiations. Other behaviours associated with departure were unrelated to recruitment, suggesting that ‘travel hoos’ facilitated joint travel. Crucially, ‘travel hoos’ were more often produced in the presence of allies than other individuals, with high rates of recruitment success. We discuss these findings as evidence for how motivation to perform a specific social activity can lead to the production of a vocal signal that qualifies as ‘intentional’ according to most definitions, suggesting that a key psychological component of human language may have already been present in the common ancestor of chimpanzees and humans.

## Introduction

In a recent comprehensive review of animal travel, Boinski and Garber concluded that group movement “…is as much a social behaviour as it is an ecological response to the distribution and availability of resources and risks” [1] (p. 680). We subscribe to this view with a study on the social dimension of group travel in chimpanzees. Wild chimpanzees often travel in small subgroups, and this requires individuals to engage in coordination and communication [2]. Travel therefore makes a promising context for investigating the social awareness available to individuals during this joint activity [3]. From previous work with chimpanzees it is already relatively well established that, to some extent, chimpanzees can take their audience into account during call production, particularly in contexts of aggression [4], sex [5], feeding [6], when encountering group members [7], and when discovering dangers [8]. Similar findings have also emerged from closely related bonobos ( *Pan* *paniscus* ). In one study, female bonobos engaging in sexual behaviours with high- (but not low-) ranking partners advertised this fact with ‘copulation’ calls [9]. These and other findings have led to the suggestion that great apes are able to adjust signal production to their surrounding audience in seemingly strategic ways. This is relevant because it suggests that the common ancestor of modern humans and the two *Pan* species might already have had some control over vocal production by taking into account the audience and the social implications of call production.

There is little doubt that chimpanzees, as well as many other primates and non-primate species, can engage in communal acts with potentially different roles, such as group hunting [10]. Another relevant example of a communal act in chimpanzees is food sharing, which mostly consists of field observations of individuals tolerating others’ scrounging on food that they control, known as ‘passive’ sharing. Actively handing a piece of food to another individual, or ‘active’ sharing, is much rarer [11]. Related experimental evidence comes from captive bonobos, who will unlock a door to let another individual into the same room in order to share food [12]. Both chimpanzees and bonobos produce food calls when discovering a new food source, sometimes also to newly arriving individuals who have not yet been feeding in the tree. This apparent vocal recruitment has been interpreted as an invitation for the recipient to feed jointly with the caller [2,6]. Whether this is to merely avoid aggression in a potentially competitive situation [13] or to actively inform them in an altruistic way is currently unclear and the topic of ongoing research. In sum, there are a considerable number of situations in which great apes engage in joint activities, which offer as many opportunities to study the psychological bases of such behaviour.

In this study, we focused on the travel behaviour of free-ranging chimpanzees ( *Pan* *troglodytes* *schweinfurthii*) of the Sonso community in Budongo Forest, Uganda [14]. Travel represents one of the main daily activities of chimpanzees, notably to find food sources, but also to reach to nesting sites or to interact with neighbours. Travel typically occurs in parties of varying sizes, often without interruption for several kilometres as if pursuing a goal [15]. Travelling with others is likely to be adaptive due to the potential dangers of encountering predators or males of neighbouring groups, which can have fatal consequences especially for single individuals [16]. Although intergroup encounters have been observed at territory borders, Sonso males do not show much ‘patrolling behaviour’, as described for other communities. Rather, they appear to control their territory by adopting foraging patterns and choosing travel routes that include the peripheral areas of their range [15]. Joint travel, in other words, is particularly important in this community because of the dangers of being in the more peripheral area.

**Figure 1:**
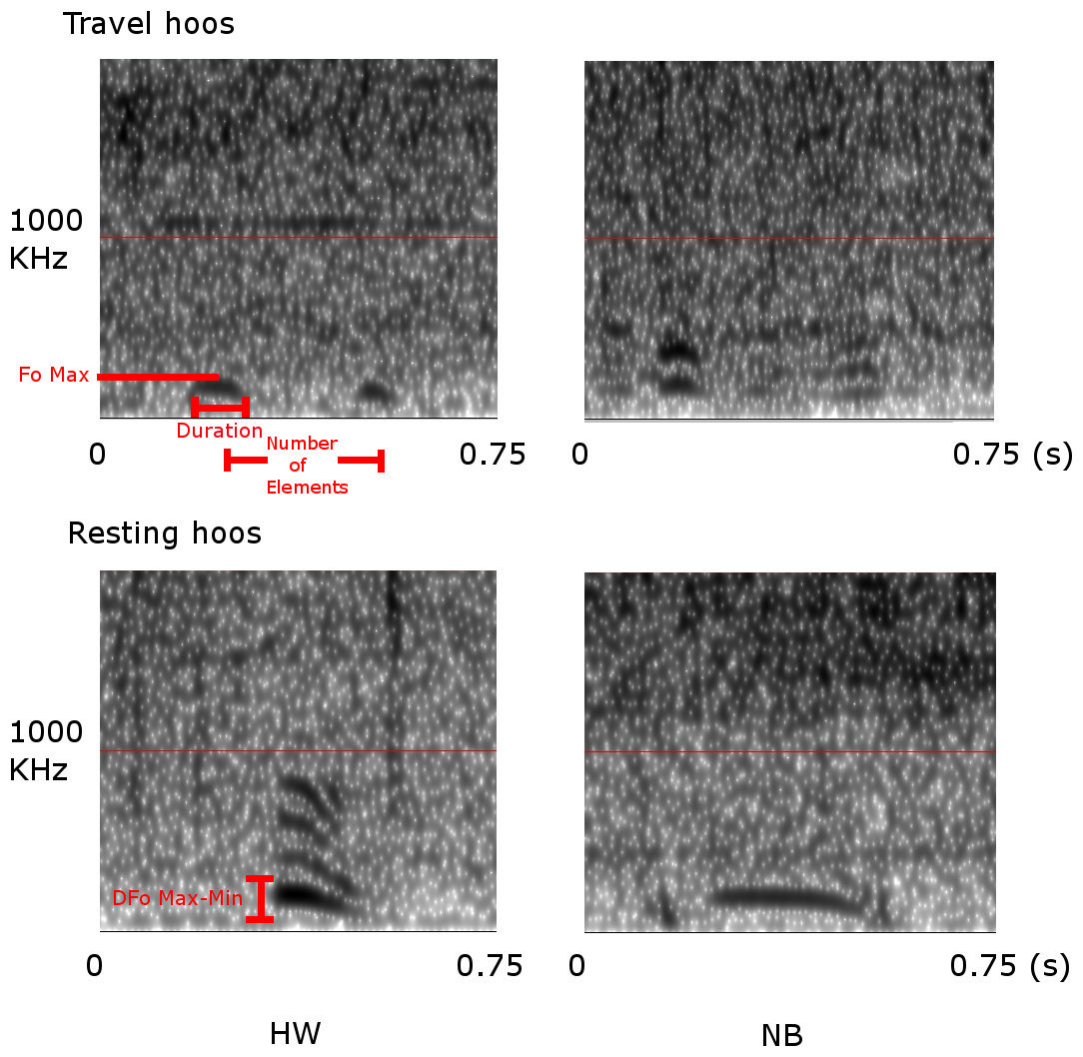
‘Hoo’ spectrograms obtained from an adult male (HW) and female (NB) of the Sonso community. Above: ‘hoos’ given during travel events (‘travel hoos’); below: ‘hoos’ given during resting events (‘resting hoos’). Compared to ‘resting hoos’, ‘travel hoos’ are significantly shorter (0.125s vs. 0.336s, t-test, N=20, t=4.455, p=0.001), have a lower maximum fundamental frequency F_0_ (178.83Hz vs. 220.47Hz, t-test, N=20, t=3.139, p=0.006), are less modulated (difference D between F_0MAX_ and F_0MIN_: 37.17Hz vs. 89.23Hz, t-test, N=20, t=3.796, p=0.001), and consist of more elements (mean 2.7 vs. 1.0, t-test, N=20, t=-3.042, p=0.014). Analyses were based on N=20 calls (N=5 travel hoos, N=5 resting hoos recorded from HW and NB, respectively).

We have observed that, in the travel context, chimpanzees produce a brief and inconspicuous vocalisation, the so-called ‘travel hoo’, which is acoustically distinct from ‘hoos’ produced in other contexts (Figure 1) [2]. Pilot observations showed that travel initiations were usually accompanied by various non-vocal behaviours, particularly ‘waiting’ and ‘checking’ (see methods), suggesting that the initiator may be expecting others to follow. To investigate whether ‘travel hoos’ function in recruiting others for a joint activity, namely group travel, we analysed the production of ‘travel hoos’ across the various stages of a travel event. We were specifically interested in whether callers directed these signals at specific audiences and how their vocal behaviour was integrated in their wider recruitment efforts.

Researchers interested in signals that function to influence others’ social behaviour and are putatively intentionally produced usually look for a range of accompanying behaviours, such as: (1) audience checking: the signaller monitors the state of attention of a recipient; (2) response waiting: the signaller pauses after producing the signal to wait for a behavioural response; (3) persistence: the signaller repeats the signal or produces a new one if the recipient’s response is unsatisfactory [17,18]. We predicted that, if ‘travel hoos’ functioned to recruit others, they should occur in situations when the caller was with others rather than alone and was actively attempting to recruit others to follow. We further predicted that, if ‘travel hoos’ functioned to initiate travel, they should also be consistently given before the locomotor behaviour occurred during the travel ‘initiation phase’, so as to alert others to forthcoming departure. We also predicted that if ‘travel hoos’ were socially directed to recruit specific group members, they should be produced preferably in the presence of a desirable audience. According to the current literature, the following classes of individuals should be particularly desirable travel partners: (i) individuals with whom the focal animal maintained strong bonds, i.e., ‘allies’ [8]; (ii) higher-ranking individuals, who might offer protection and other social benefits [9,19] and, in the case of males; (iii) oestrous females potentially available as mating partners [20]. In contrast, if ‘travel hoos’ were a reflection of a more general motivation to travel, and if individuals did not expect a specific answer from the audience, then they should be delivered randomly throughout the ‘initiation phase’. Similarly, ‘waiting’ and ‘checking’ behaviours should not necessarily follow call production.

## Materials and Methods

### Ethics statement

This study was observational and non-invasive. The research proposal was approved by the Ethics Committee of the School of Psychology at the University of St Andrews. Permission to work in the Budongo Forest was obtained from the Ugandan National Council for Science and Technology (UNCST), the Uganda Wildlife Authority (UWA) and the National Forestry Authority (NFA) after review and approval of the research proposal.

### Study site and subjects

The study was carried out in the Budongo Forest Reserve in western Uganda, at the edge of the western Rift Valley along Lake Albert (latitude 1° 37'-2° 00'N; longitude: 31° 22'-31°46'E). The reserve has a size of 793 km^2^, which consists of moist, semi-deciduous tropical grassland and 428 km^2^ of forest [21,22]. The forest contains approximately 640 chimpanzees, about 8-10 communities overall, with a density of 1.36 individual/km^2^ [23]. At the beginning of the study, the Sonso community consisted of 74 individuals; 21 adult females, 20 adolescent females, 12 infant females, 9 adult males, 8 adolescent males and 4 infant males. Two infants were born during the study while 3 adults died from old age or injury after being caught in a mantrap (a powerful spring mechanism designed to capture or seriously injure large animals). The home range of the community has been estimated to be 6.78 km^2^ [24]. Data were collected during two field seasons (January 17^th^ to March 25^th^, 2009; September 3^rd^, 2009 to September 3^rd^, 2010) from 33 individuals (N=15 males, aged 8 to 49; N=18 females, aged 12 to 47). Data collection was based on focal animal sampling [25] by following subjects on their daily travels from 07:00 to 16: 00.

### Definitions

#### Travel events

We defined ‘travel’ as an event that began with the termination of a non-locomotion activity, followed by locomotion of at least 10m, and ended with the commencement of a non-locomotion activity, usually feeding, grooming or resting. Although locomotion was sometimes interrupted, we considered it part of the same travel event provided the interruption was less than 5 minutes and did not lead to other activities. We only analysed travel events that started on the ground, as it was not possible to reliably document ‘hoo’ calls given within trees.

#### Travel phases

Travel events consisted of three distinct phases, starting with an ‘initiation phase’, defined as the period between cessation of the previous activity and the beginning of the subsequent ‘movement phase’. The ‘initiation phase’ was especially critical for our analysis and typically lasted for about one minute. The subsequent ‘movement phase’ was defined as a locomotor sequence of at least 10m leading the focal animal out of sight of the position of its previous non-locomotion activity. A travel event was terminated by the ‘arrival phase’, defined as the period during which the focal animal stopped moving and initiated a non-locomotion activity for at least 5 minutes. ‘Travel hoos’ could be produced by any individual participating in a travel event and could occur during all three phases. We also scored the general context during the production of each travel hoo, as defined in table 1.

**Table 1:** Travel events and context of ‘travel hoos’ recorded from focal individuals between January 2009 and September 2010.

Situation	Travel phase	Description	Presence of Wait /Check	Vocal events	Silent events
Initiating	I	Focal interrupts current activity and starts moving	Yes	91	137
Recruiting	I, M	Focal is already travelling and produces recruiting behaviours to others not yet travelling	Yes	87	1
Following	I, M	Focal follows another individual that initiated a move or recruited the focal while travelling	No	28	34
Joining	M, A	Focal joins a group that is already performing an activity that is not travel	No	21	9
Vocalising while travelling	M, A	Focal produces a vocalisation during travelling	No	24	n.a.
Replying	I, M, A	Focal produces a vocalisation in response to another individual’s vocalisation	No	16	n.a.
Unknown	I, M, A	Travel event could not be classified with certainty	n.a.	8	0
Total				275	181

Non-vocal travel events are also listed for comparison.

I: initiation phase; M: movement phase; A: arrival phase. Wait: The focal animal stands motionless on all four limbs for at least 5s. Check: The focal animal gazes backwards, seemingly at one or more individuals (see table 2).

#### Key behaviours

Focal animals could play two distinct roles during a travel event: either they were ‘initiators’ or ‘followers’. For both initiators and followers, we coded a number of key behaviours, which could occur during the different phases of a travel event (table 2; see video S1). In particular, we were interested in behaviours that suggested that the focal animal was monitoring its audience and/or waiting for specific responses. Three behaviours appeared to fulfil these criteria: ‘hooing’, ‘waiting’ and ‘checking’ (table 2).

**Table 2:** Key behaviours produced by focal animals during the different phases of a travel event (see video S1).

Behaviour*	Travel phase	Definition
*Initial gazing*	I	Focal animal stares horizontally into the forest for at least 5s
*Waiting*	I, M	Focal animal stands motionless on all four limbs for at least 5s
*Checking*	I, M	Focal animal gazes backwards (usually between 90 and 180 degrees, relative to the general travel direction), in the direction of one or more individuals
*Hooing*	I, M, A	Focal animal produces a low-intensity, low-pitched (100-200Hz), voiced utterances consisting of 1 to 3 unmodulated 100-200ms brief elements with descending fundamental frequencies (F_0_) of around 200Hz, 150Hz and 100Hz (see Figure 1)
*Moving*	I, M, A	Focal animal moves along the general travel direction

Direction of ‘initial gazing’ and ‘moving’ were scored as deviations from the geographic north in roughly 10-degree intervals. * If one of the behaviours occurred repeatedly, we only coded the first appearance for each initiation phase. I: initiation phase; M: movement phase; A: arrival phase.

#### Social factors

To investigate the impact of social factors on behavioural decisions during a travel event, we determined the social bonds and relative ranks of the focal animals and, for adult females, their relative stage of fertility. We used social bond data determined by another study conducted during the same time period [8] (see Data S1). We calculated dominance ranks from pant-grunting behaviour, a reliable indicator of rank relations in chimpanzees [7]. For the females, pant-grunting between individuals was not so common, but it was still possible to group individuals into three rank classes: ‘high’, ‘middle’ and ‘low’. Individuals who received pant-grunts from at least four other females were classed as high-ranking. Individuals who received pant grunts from 1-3 other females were classed as middle-ranking, and individuals who never received pant-grunts were classed as low-ranking. For the males, it was possible to determine individual dominance ranks, mainly because they interacted more often with each other and produced pant grunts more often than the females [7]. As in other communities, all adult males were dominant over all adult females, including the alpha female. Finally, we scored all adult females for the presence and size of their sexual swellings (stages 0-4). A female was considered to be ‘in oestrous’ if her swelling surpassed stage 3 [26].

### Statistical methods

To analyse the sequential organisation of behaviours seen during the ‘initiation phase’, we used a Friedman test with the order of appearance of each of the five behaviours, the behaviour type, and the identity of the individuals as variables. To control for the identity of the focal animals and their different contributions to the dataset (individuals contributed 1 to 11 initiation phases with an average of 3.54 sequences), we entered the average order of appearance of each behaviour per individual. We used post hoc Tukey-tests for pairwise comparisons.

To analyse the effect of the presence of other group members on vocal production, we ran a Generalized Linear Mixed Model (GLMM) comparing vocal and non-vocal travel events during solitary and social travel. Call production was entered as the dependent variable coded as a binomial response, the presence of an audience as a fixed factor and the identity of the focal animals as a random covariate (as individuals contributed several data points). For all travel events with an audience, we checked if ‘travel hoo’ calls were more likely to be given if a preferred individual was in the audience. To this end, we first calculated, separately for each individual, the frequency of vocal and non-vocal events with an ally, high-ranking individual or oestrous female nearby. We then ran a paired t-test across all individuals for whom we had collected at least three observations in each of the two relevant conditions (presence and absence of the potentially desirable partners). For example, to be included in the analysis on allies, an individual would have to be observed in three different independent travel events both with and without an ally present. We then ran a GLMM with the production of travel hoos as the dependent variable coded as a binomial response. The sex of the focal animal, the presence of a dominant individual and the presence of an ‘ally’ were included as fixed factors. Females in oestrous were not included here, because their status as desirable travel partners for other females was unclear. As previously, the identity of the focal animal was entered as a random covariate.

To investigate whether travel hoos led to recruitment of other individuals, we continuously estimated the distance of all audience members to the caller in metres. Successful recruitment was scored if at least one individual followed the initiator of a travel event. We calculated, separately for each individual, the frequency of vocal and non-vocal cases in which the focal animal was successful in recruiting at least one other individual and compared the two conditions with a paired t-test across all individuals for which we had at least three independent vocal and three independent non-vocal initiations.

Secondly, we tested whether ‘wait’ and ‘check’ were associated with vocal or non-vocal travel events using a paired t-test analysis. Similarly, we only included individuals for whom we had displays of the relevant behaviour (‘wait’ or ‘check’) in at least three independent vocal and three non-vocal initiations. We then ran a GLMM, with recruitment success as the dependent variable (coded as a binomial response). Presence of ‘travel hoos’, ‘wait’ and ‘check’ behaviours and the sex of the focal animal were entered as fixed factors. The identity of the focal subject was entered as a random covariate.

Finally, we analysed all cases in which the caller was successful in recruiting others for travel by checking if allies were significantly more common amongst the recruited individuals than expected. To do so, we used a GLMM, with the rate of successful recruitments of allies as the dependent variable coded as a binomial response, and the presence of a call as the fixed factor, provided that at least one ally was in the audience. The identity of the focal subject was entered as a random covariate.

All statistical tests used in the analysis were calculated with SPSS 19.0 and were two-tailed.

## Results

### Travel hoos are used to initiate departure

While following 33 different focal animals, we recorded a total of N=456 travel events. N=275 (60.3%) included at least one ‘travel hoo’, while the remaining N=181 events (39.7%) were silent (table 1). Travel hoos were mainly given by individuals trying to lead (N=178 total, 64.7% of cases, table 1), either by initiating (N=91) or recruiting (N=87, as defined by the presence of ‘wait’ or ‘check’). Hoos were also given in response to other individuals producing hoos (N=16) or for unknown reasons during travel (N=24). Finally, some hoos were produced when the focal subject was following another individual (N=28) or when joining a group (N=21, table 1).

**Figure 2:**
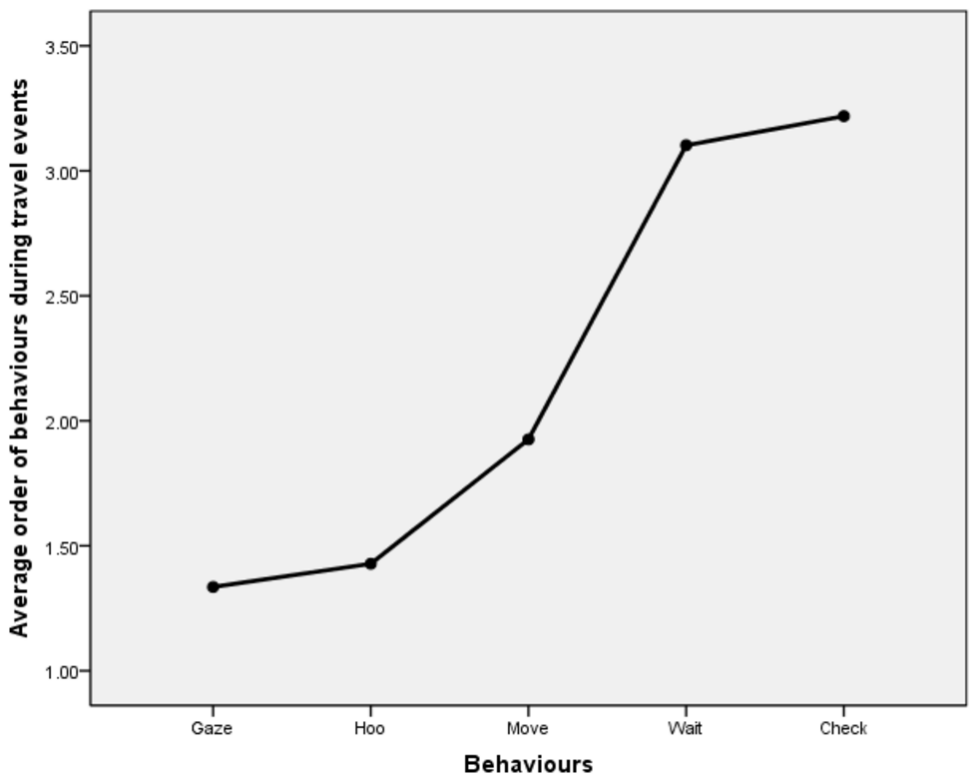
Mean plot showing the sequential order of behaviours observed during travel events that included at least one ‘travel hoo’. ‘Initial gazing’ and ‘hooing’ (p=0.975), and ‘waiting’ and ‘checking’ (p=0.971), were not significantly different from each other, but differed from ‘initial moving’ (p=0.002, p=0.015, p<0.001 and p<0.001, respectively, Tukey HSD pairwise comparisons).

For 85 of 91 ‘initiation phases’ initiated by travel hoos (N=24 individuals), we were able to document the order in which the five key behaviours related to travel (table 3) were produced. Order was non-random (Friedman test, N=105, df=2, F_r_=20.130, p<0.001, Figure 2), with ‘initial gazing’ (N=85) typically shown first, followed by production of ‘travel hoos’ (N=85), ’initial moving’ (N=85), ‘waiting’ (N=49), and ‘checking’ (N=33). In pairwise comparisons, the mean order in the sequence of ‘initial gazing’ and ‘hooing’ (p=0.975) on the one hand, and ‘waiting’ and ‘checking’ on the other hand (p=0.971), were not significantly different from each other, respectively, but differed from ‘initial moving’ (p=0.002, p=0.015, p<0.001 and p<0.001, respectively; Tukey HSD tests, table 3). ‘Hooing’ before ‘initial moving’ occurred in 51 of 85 cases (60.0%); ‘hooing’ at the moment of ‘initial moving’ occurred in 8 of 85 cases (9.6%).

**Table 3:** Average order of appearance of the five key behaviours during the initiation phase.

Behaviour	N	Average order	
		Total	Average
Gaze	85	1.39	1.34
Hoo	85	1.46	1.43
Move	85	1.96	1.93
Wait	49	3.10	3.10
Check	33	3.06	3.22

Total: based on the total N=85 sequences found across individuals; Average: based on the average order of each behaviour per individual.

The average delay between the end of ‘hooing’ and ‘initial moving’ was 4.23s (N=55). When ‘initial moving’ preceded ‘hooing’, travel hoos were produced on average 2.70s (N=20) after departure.

For N=111 vocal and non-vocal travel events, we were able to compare the direction of the ‘initial gaze’ and the direction of the subsequent ‘initial move’. The two directions differed in only 8 of 111 cases, while coinciding in the remaining 92.3% of cases.

### Travel hoos are produced to recruit allies

In order to assess the impact of nearby listeners on call production, we compared all travel events with and without hoos but excluded cases where the focal individual was alone or alone with dependent offspring. For the vocal travel events, we excluded 12 of 275 cases in which the focal individual was alone (N=2 cases, 0.7% of the total number of cases) or alone with dependent offspring (N=10 cases). For the non-vocally induced travel events we excluded 51 of 181 non-vocal travel events because the focal animal was alone (N=32 cases) or alone with dependent offspring (N=19 cases), which resulted in a final sample size of N=263 vocally initiated travel events (males: N=162; females: N=101; 66.9% of N=393 total) and N=130 non-vocally initiated travel events (males: N=70; females: N=60, 33.1% of N=393 total). We found a significant difference between the number of cases excluded in non-vocal and vocal travel events (GLMM, Estimate=2.092, Standard Error (S.E.)=0.344, t=6.086, p<0.001), showing that travel events included a ‘travel hoo’ most often in the presence of an audience.

171 of the 263 vocal events (65.2%) were single entries to the dataset (i.e. no more than one event per day per individual). 92 of 263 (34.8%) vocal travel events were multiple entries from individuals that had been recorded more than once on the same day. 60 of these 92 vocal events were given during different travel events with different audiences. The average interval between two recorded travel events was 84.59 min (N=32, range 10–279 min). In the shortest case, the focal animal stopped travelling to feed on a hard-shelled fruit found on the ground but initiated another travel event when he saw his mother approaching 10 min later. 14 of 92 vocal events were considered to belong to the same travel event but were given to different audiences (i.e. the caller was already engaged in a travel bout, but called again during the same event when other chimpanzees had joined the party). Finally, 18 of 92 vocal events were given by the same individual and during the same travel event (9 different events total) and to the same audience and the 9 repetitions were thus classified as 'persistence'. In these cases, the caller was unsuccessful in recruiting others the first time and re-launched his or her efforts shortly thereafter (N=9, mean=3.80 min, range 0–13 min).

94 of the 130 non-vocal events (72.3%) were single entries to the dataset (i.e. no more than one event per day per individual). 36 of 130 (27.7%) travel events were multiple entries from individuals that had been recorded more than once on the same day. 26 of these 36 cases were different travel events with different audiences. The average interval was 79.19 min (N=16, range 5–280 min). In the shortest case, the focal animal travelled with one individual, engaged in grooming with him for 5 minutes, then initiated another travel bout when a female appeared. Out of the 10 remaining events, 8 appeared to be different travel bouts (separated by an average of 50.50 minutes, N=4, range 28–78 min) but were recorded with the same audience. Finally, the last two cases occurred consecutively with the same audience and the second case was thus classified as persistence.

We then investigated if members of key social categories, i.e. allies, dominant individuals, or oestrous females, had an influence on the production of ‘hoos’. Allies were present in 225 of 393 travel events, with calls given in 170 of 225 cases (75.6%). In contrast, allies were absent in 168 of 393 cases, with calls given in 93 of 168 cases (55.4%). We were able to include the data from 14 individuals (8 males and 6 females) with at least three independent events in the ‘ally present’ and ‘ally absent’ conditions (N=212 vocal events; N=101 non-vocal events), and found that these individuals called significantly more often when an ally was present in the audience (paired t-test, t=3.374, df=13, p=0.005, table 4).

**Table 4:** Ratio of vocal and silent travel events with different audiences.

Audience	Travel hoo	Silent	Total
Female in swelling absent	67.9	32.1	140
Female in swelling present	72.8	27.2	92
Excluded (Female caller)	63.1	36.9	161
Ally present	75.6	24.4	225
Ally absent	55.4	44.6	168
Dominant present	69.9	30.1	266
Dominant absent	60.6	39.4	127
Total	66.9	33.1	393

Total: number of events in each case.

Dominant individuals were present in 266 of 393 travel events, with calls given in 186 of 266 cases (69.9%). In contrast, dominant individuals were absent in 127 of 393 cases, with calls given in 77 of 127 cases (60.6%). We were able to include the data from 11 individuals (6 males and 5 females) with at least three independent events in the ‘dominant present’ and ‘dominant absent’ conditions (N=178 vocal events; N=84 non-vocal events), and found that these individuals did not call significantly more often when a dominant individual was present in the audience (paired t-test, t=0.734, df=10, p=0.48, table 4).

Oestrous females were present in 92 of 232 travel events initiated by males, with calls given in 67 of 92 cases (72.8%). No oestrous female was present in 140 of 232 cases, with calls given in 95 of 140 cases (67.9%). We were able to include the data from 9 males with at least three independent events with oestrous and non-oestrous females present (N=151 vocal and N=67 non-vocal events), and found that these individuals did not call significantly more often when an oestrous female was present in the audience (paired t-test, t=-0.234, df=8, p=0.821, table 4).

Finally, when simultaneously assessing the effects of allies and dominant individuals on call production, we found a strong effect for the presence of allies (GLMM, Estimate=0.838, S.E.=0.229, t=3.668, p<0.001) but not for dominant individuals (GLMM, Estimate=0.400, S.E.=0.244, t=1.636, p=0.103), regardless of the focal animal’s sex (GLMM, Estimate=0.233, S.E.=0.241, t=0.970, p=0.333), and no intercept (GLMM, Estimate=-0.277, S.E.=0.287, t=-0.966, p=0.335).

### Travel hoos enhance rates of successful recruitment

To assess the recruiting power of hoos, we compared initiation events with or without hoos. We excluded 62 cases in which the focal individual was alone or with dependent offspring, which resulted in a final sample size of 166 travel events. 77 of 166 events (46.4%) were initiated by hoos, while 89 cases (53.6%) were silent departures, with hoos sometimes given at later stages during travel. 55 of 77 (71.4%) vocally initiated travel events led to a travel party (two or more individuals, including the travel initiator), compared to 30 of 89 non-vocally initiated travel events (33.7%). We were able to include 11 individuals (6 males and 5 females) with at least three independent vocal events (N=60) and non-vocal events (N=61). Focal individuals were significantly more likely to obtain a successful recruitment when calling than when remaining silent (paired t-test, t=3.805, df=10, p=0.003).

‘Checking’ was recorded in 39.0% and ‘waiting’ in 58.4% of vocally initiated events (N=77), compared to 25.8% and 53.9% of silent events (N= 89). We were able to include 11 individuals (6 males and 5 females) displaying ‘waiting’ behaviour in at least three independent vocal events (N=62) and non-vocal events (N=66), and found no significant difference between vocal and non-vocal events (paired t-test, t=1.935, df=10, p=0.082). We were able to include 13 individuals (7 males and 6 females) displaying ‘checking’ behaviour in at least three independent vocal events (N=67) and non-vocal events (N=78), and found significantly more ‘checking’ in vocal than non-vocal events (paired t-test, t=2.249, df=12, p=0.044).

**Figure 3:**
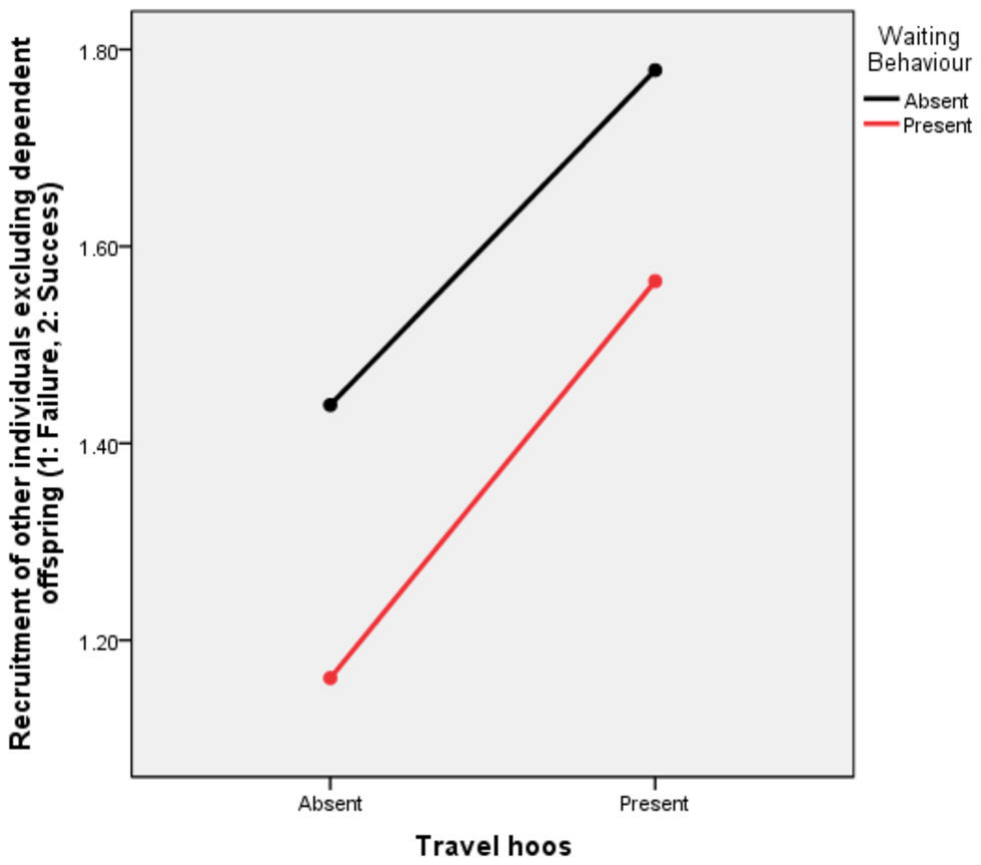
Profile plot showing the successes of focal individuals in recruiting other individuals as a function of the presence of ‘hooing’ and ‘waiting’. The production of ‘hoos’ had a significantly positive effect on recruitment (GLMM, t=4.857, p<0.001), while the presence of ‘waiting’ had a significantly negative effect (GLMM, t=-2.457, p=0.015).

When comparing successful and unsuccessful recruitment events, focal individuals were significantly more likely to be successful if they produced a travel hoo than if they remained silent (GLMM, Estimate=1.824, S.E.=0.376, t=4.857, p<0.001). However, individuals were significantly less likely to wait if they had already been successful in recruiting another individual (GLMM, Estimate=-1.085, S.E.=0.442, t=-2.457, p=0.015). Checking behaviour was not affected in the same way (GLMM, Estimate=-0.313, S.E.=0.480, t=-0.653, p=0.515) and the focal animal’s sex also had no effect (GLMM, Estimate=-0.183, S.E.=0.359, t=-0.509, p=0.611), with no intercept (GLMM, t=-0.682, p=0.496; Figure 3).

### Allies’ responses to travel solicitations

In a final analysis, we investigated whether, in the cases where allies were present in the audience when a call was produced, they were among the recruited individuals. Allies were recruited in 66 of 101 vocal travel events (65.3%, including N=18 cases in which no one joined the caller). In comparison, allies were recruited in 13 of 37 non-vocal travel events (35.1%, including N=22 cases in which no one joined the caller), a significant difference (GLMM, Estimate=1.102, S.E.=0.419, t=2.630, p=0.010).

## Discussion

### Function of travel hoos

Travelling is a goal-directed behaviour that usually involves several individuals coordinating their activities and goals. In line with this, we observed chimpanzees monitoring the effect of their departure on others by displaying ‘waiting’ and ‘checking’ behaviour. One possible interpretation is that chimpanzees are aware that their departure influences other individuals by interrupting a current activity in favour of joint travel. Our data show that call production enhances the likelihood of recruiting followers. We did not observe any obvious signs of gestural communication in this context, although we cannot rule out the presence of more subtle signals.

We found that call production was most common when other group members were occupied with other activities during the ‘initiating’ and ‘recruiting’ contexts (table 1). In these circumstances we also found ‘waiting’ and ‘checking’ behaviours (table 2), suggesting that the caller was monitoring the effect of its calls and own locomotor behaviour on the audience. The subjects typically produced travel hoos before they showed ‘initial moving’ and monitoring behaviours (‘wait’ and ‘check’), suggesting that the calls function to signal an impending departure. Travel hoos were nearly always produced to unrelated individuals or paternal half-siblings and very rarely when travelling alone or when travelling with dependent offspring only. During the entire study, there were only two instances in which travel hoos were given in the absence of an audience, both times by a critically ill adult male (BB) shortly before his death, but here the calls may have been directed at the observer (TG) and his field assistant. Travel hoos were given preferably if allies were in the vicinity, while the presence of dominant individuals or female in oestrous (for males) had no effect. In other words, chimpanzees prefer to travel with individuals with whom they maintain affiliative bonds, as expressed in grooming interactions and spatial proximity during resting.

Regarding the effectiveness of the calls, we found that travel initiations were more successful in recruiting others if they contained travel hoos than if they were silent. Moreover, recruitment was not due to other behaviours common to departure, such as gazing in the direction of the forthcoming travel as this behaviour happened in both vocal and non-vocal events, although this may additionally inform recipients about the direction the caller wishes to go towards. Another common behaviour was ‘waiting’ (58% of vocal and 53% of non-vocal departures included ‘waiting’) although this behaviour was associated with lower recruitment success (Figure 3). Typically, recruitment happened almost instantly, so that ‘waiting’ may be more a consequence of unsuccessful recruitment attempts. In unsuccessful initiators, we found signs of ‘persistence’ in the form of repeated ‘checks’ and ‘hoos’, but low sample size prevented more systematic analyses. Chimpanzees were generally highly successful in their recruitment efforts. However, we found significantly more ‘checking’ in vocal than silent initiations, suggesting that callers were monitoring the effect of their calling on the nearby audience. Finally, we found that when travel hoos were produced in the presence of allies, they were usually amongst the recruited individuals, suggesting that callers produced hoos for closely affiliated individuals to follow.

A last relevant observation was the lack of sex differences in vocal production or recruitment success. This shows that females, despite their reputation of being less social than males [27,28], are also motivated to engage in joint travel, suggesting that social interactions in female chimpanzees may be equally complex [29].

### Are travel hoos intentional vocal signals?

Our findings bear some resemblance to a growing literature that interprets ape gestures as intentional signals [17,18,30,31]. Although there is no generally accepted definition for intentionality in animal communication, most authors agree on certain criteria. A signal is usually considered ‘intentional’ if the signaller targets a specific recipient ‘with the aim of’ modifying its behaviour [17,18,30,31]. Such cases are usually contrasted with communication as ‘involuntary expressions’ of internal states over which the individual has no apparent control. The main behavioural criteria to identify the intentional nature of a signal are: ‘audience checking’, ‘response waiting’ and ‘persistence’ [17,18,30,31]. Using these criteria, our results suggest that travel hoos qualify as intentional signals. Intentional signals, in other words, are not limited to the gestural domain, as often claimed, but can include vocal signals, at least in great apes. We consider this an important finding because it suggests that animals may have more control over their vocal production than previously thought [32].

### Alternative hypotheses

It is essential to remain cautious when considering possible psychological states underlying behavioural data. For example, a recent study on grouper fish reported behavioural patterns consistent with definitions of referential communication in primates and corvids [33,34] although they were unlikely to be the result of advanced cognitive abilities [35]. An alternative way of interpreting our findings is in terms of a combined motivation to travel and to vocalise in the presence of an ally. Hormones may also play a role during such processes, as the presence of allies has a demonstrated effect on the hormonal profile in wild chimpanzees [36]. Nevertheless, it may not be possible to explain call production by these two factors alone, as demonstrated by the fact that travel initiators remained silent in 25% of cases when allies were nearby. This observation, although not excluding potential ecological influences on call motivation (for instance related to the targeted area or resource), shows nonetheless a flexibility of control on ‘hoo’ production unlikely to be explained by hormonal factors alone.

A potentially parsimonious stance is that ‘travel hoo’ production is motivationally governed (“I am in the mood for travelling…”), but that call delivery is modulated by cognitive assessments of the ongoing context. For example, the presence of an ally may trigger a desire to travel jointly, so that call production takes on a goal-directed element and becomes more intentional (“I am in the mood for travelling with you”). A second interpretation of the call could be “I am in the mood for travelling with someone”, but again, the call production itself would still be intentional: in effect, individuals can make public their motivation to travel through their control over the production of ‘travel hoos’, depending on their assessment of the surrounding audience.

Currently, it is hard to decipher between the two alternatives, notably because ‘motivation’ and ‘intentionality’ are notions that are empirically hard to access, especially when working with wild animals. Nevertheless, we have found evidence for key criteria used to identify intentional communication, notably audience checking, response waiting and persistence. We acknowledge that such criteria are rather superficial because they are behaviourally bound, and could be interpreted in a motivational perspective. Such scepticism may be justified, and some claims of intentionality in animals but also in pre-linguistic infants may have to be revised in light of stronger data, notably coming from the field of neurobiology where both the perception of others as intentional beings [37] or the production of calls [38] can be studied. Before such data are available, however, progress can be expected by carefully designed field playback experiments, perhaps in conjunction with data on physiological variables.

## Supporting Information

Video S1 **Individual ZM, while leaving a Ficus *sur* feeding tree, displays the waiting behaviour at 00:07, and produces travel hoos at 00:17.** She recruits individual MK, who displays the checking behaviour at 00:22.
(WMV)

Data S1 **List of ally relationships in the Sonso community for the period 2009-2010.**
(PDF)
