# Supplementary material for: Vocal Recruitment for Joint Travel in Wild Chimpanzees
Source: PLoS One. 2013 Sep 25;8(9):e76073. doi: 10.1371/journal.pone.0076073 (PMC3783376; doi:10.1371/journal.pone.0076073)
Supplement: Data S1 — List of ally relationships in the Sonso community for the period 2009-2010. (PDF) [file pone.0076073.s002.pdf]

List of Ally Relationships in Sonso for the Period 2009 - 2010

ID	Jan-Mar09	Oct-Dec09	Jan-Mar10	Apr-Jun10
nk	kt, ku, ml, nb, rh, wl, zf	ht, jl, nb, rh, tk, zf	jl, kl, kw, ml, zf	kl, ms, nb, zm
zf	kz, nk, zm	nk, sq	kt, ms, nk	hw
ms	mk, nb, sq	mk, nb	nb, zf	kw, nb, nk, zl
sq	fd, hw, ml, ms, nr, sm, zl	fd, fk, nb, re, zf, zl	hw	be, fd, fk, hw, kz
tk	none	hw, nk	n.a	n.a
hw	ml, sq, zl	ht, jl, kt, kz, mk, nr, re, tk	ht, jl, kt, nr, ps, sq, re, vr, zl	fd, kw, ml, re, sq, zf
kt	ku, ml, nk	an, fd, hw, ku, wl	fd, hw, ku, ml, zf, zg	jl, nb, zm
fd	fk, nr, sq	fk, fl, kt, sm, sq	fl, jl, kt, sm, zl	fk, hw, sq
sm	nr, se, sq	fd, kz, mk, se	fd, se, wl	kz, zl
zl	hw, nr, mk, sq	jl, jn, mk, ml, nr, rs, sq	fd, hw, jl	ms, sm, zd
kz	kw, zf	hw, jl, kw, sm	kw	kw, sm, sq
fk	fd	fd, fl, mk, sq	fl	fd, fl, sq
ps	none	nr	hw	none
nb	kl, kw, ms, nk, nr	kl, ms, nk, nr, sq	ms	kl, kt, kw, ms, nk, nr, zm
nr	fd, nb, sm, sq, zl	hw, nb, ps, zl	hw	nb
kw	kl, kz, nb, sb	kz	kz, nk	hw, kl, ku, kz, ms, nb
ku	kt, nk	kl, kt	kt	kw
kl	kw, nb	ku, nb	kw, nk	kw, nb, nk
zm	zf	none	zg	kt, nb, nk
ky	sb	none	none	sb
rh	nk	nk	none	none
rs	none	zl	none	none
ml	hw, kt, nk, sq	wl, zl	hw, re, wl	hw, re, wl
jn	none	none	none	none
mk	ms, re, se, zl	hw, kz, ms, sm, zl	none	re
wl	nk	kt, ml, re	re, sm	ml, ok
re	mk	hw, mk, sq, wl, zg	hw, wl	hw, mk, ml, zg
se	mk, sm	sm	sm	none
sb	be, kw, ky	none	none	ky
be	sb	none	no data	sq
fl	none	fd, fk	fd, fk, ml	fk
bt	none	no data	no data	no data
ht	none	nk, hw	hw	none
jl	none	hw, kz, nk, zl	fd, hw, nk, zl	kt
vr	none	re	hw	none
kg	none	no data	no data	no data
zg	no data	re	kt, zm	re
zd	no data	none	none	zl
ok	no data	no data	no data	wl
gl	no data	none	no data	no data
tj	no data	none	none	none
an	no data	kt	none	none

Jan-March 2009

bCRI	nk	zf	ms	sq	tk	hw	kt	fd	sm	zl	kz	fk	ps	nb	nr	kw	ku	kl	zm	ky	rh	rs	ml	jn	mk	wl	re	se	sb	be	fl	bt	ht	jl	vr	kg
nk		14.7	-0.9	-9.1	-1.5	-4.7	6.27	-0.6	-2.3	-6.8	-2.3	-2.3	-4	31.5	0.5	16.1	16.5	-5.3	-3.6	0	11.6	0	4.68	0	0	2.99	6.44	-3.6	0	0	31.6	0	-1	2.16	0	0
zf			-4.9	-3.1	0	-1.9	0	0.54	0	-3.1	3.55	0	-3.1	22.2	-4.9	0	0	0	24	0	0	0	0	0	3.87	-3.4	0	0	0	108	0	0	0	8.66	0	0
ms				6.1	0.54	1.08	-7.3	-3.8	0	-2.7	-3.6	-2.7	0	50.3	0	4.11	0	0	-12	0	2.63	0	1.28	-8.6	5.15	0	1.22	0	0	0	0	0	-7.5	0	0	0
sq					-2.9	2.9	0	20.2	1.7	5.59	-5.9	7.93	0	-4.6	5.26	-12	0	-9.2	-14	0	0	0	4.98	0	0	0	0	0	0	0	-4.6	0	2.76	0	0	0
tk						0	0	-0.6	0	0.52	3.53	6.29	0	0	-6.2	0	0	-6.2	-20	0	0	0	0	0	0	0	11	0	0	0	0	0	0	0	0	0
hw							1.14	0	0	5.87	-2.7	0	0	0	0	-8.6	0	-4.3	-8.1	-17	0	0	9.79	0	3.87	0	0	0	0	0	0	0	0	-11	0	-4.3
kt								-5.4	-8.6	0.52	-1.2	0.63	0	-4.1	1.3	0	8.83	-4.8	-4.8	0	0	0	16.8	0	0	13.2	-9.5	-17	0	0	-9.5	0	0	0	0	0
fd									2.22	0	0	5.03	0.57	-8.7	2.59	0	0	-8.7	1.3	1.3	0	0	1.3	0	0	0	0	0	0	0	-56	0	0	0	0	0
sm										0	0	0	0	0	3.98	0	-9	0	0	0	0	0	0	0	0	0	0	41.2	0	0	0	0	0	0	0	0
zl											-1.4	0	0	-12	26	-26	-5.1	-4.7	0	-37	0	0	-36	-14	5.1	-4.1	-8.3	-4.1	0	0	0	0	-4.1	-17	0	0
kz												-3.2	4.77	-4.7	-4.7	86	-5.1	0	0	0	0	0	-9.4	-25	0	-4.7	0	0	0	0	0	0	0	0	0	0
fk													0	0	-4.3	0	0	0	-8.1	-17	-8.8	0	0	0	0	0	11.3	-18	0	0	0	0	0	0	0	0
ps														-4.6	0	0	-15	0	0	0	0	0	0	0	0	0	0	0	0	0	0	0	0	0	0	0
nb															67.3	5.73	11.2	46.2	-11	-32	0	-11	0	0	0	0	-11	0	0	0	0	0	0	0	0	0
nr																-23	-13	-11	-21	-139	-24	0	0	0	0	0	-23	0	0	0	0	0	-27	-60	0	0
kw																	0	49.6	-12	0	0	0	0	-23	0	-28	-23	5.39	26.2	0	0	0	-1.7	0	0	0
ku																		2.59	0	38.1	0	-13	0	0	0	0	1.75	0	0	0	0	0	0	0	0	0
kl																			-21	0	2.59	0	0	0	13.7	-31	0	-32	24.2	0	0	0	0	0	0	0
zm																				45.2	0	0	0	0	0	0	0	0	0	0	0	0	0	-21	0	0
ky																					0	0	0	0	0	0	38.1	0	31.4	0	0	0	0	0	0	0
rh																						3.79	0	0	0	0	0	0	0	0	0	0	0	0	0	0
rs																							0	0	0	0	0	0	0	0	0	0	0	0	0	0
ml																								5.18	0	0	27.5	0	0	0	0	0	0	0	0	0
jn																									0	0	0	0	0	0	0	0	0	0	0	0
mk																										5.57	18.4	39.5	0	0	0	0	0	0	-125	0
wl																											-5.9	0	0	0	0	0	0	14.5	0	0
re																												0	1.75	0	0	0	0	0	0	0
se																													0	0	0	0	0	0	0	0
sb																														179	0	0	0	0	0	0
be																															0	0	0	0	0	0
fl																																0	0	0	0	0
bt																																	0	0	0	0
ht																																		14.3	0	0
jl																																			21.1	0
vr																																				0
kg																																				

BIDIR	nk	zf	ms	sq	tk	hw	kt	fd	sm	zl	kz	fk	ps	nb	nr	kw	ku	kl	zm	ky	rh	rs	ml	jn	mk	wl	re	se	sb	be	fl	bt	ht	jl	vr	kg
nk		**m**	n	n	n	n	**m**	n	n	n	n	n	n	**m**	n	n	**m**	n	n	n	**m**	n	**m**	n	n	**m**	n	n	n	n	n	n	n	n	n	n
zf			n	n	n	n	n	n	n	n	**m**	n	n	n	n	n	n	n	**m**	n	n	n	n	n	n	n	n	n	n	n	n	n	n	n	n	n
ms				**m**	n	n	n	n	n	n	n	n	n	**m**	n	n	n	n	n	n	n	n	n	n	**m**	n	n	n	n	n	n	n	n	n	n	n
sq					n	**m**	n	**m**	**m**	**m**	n	n	n	n	**m**	n	n	n	n	n	n	n	**m**	n	n	n	n	n	n	n	n	n	n	n	n	n
tk						n	n	n	n	n	n	n	n	n	n	n	n	n	n	n	n	n	n	n	n	n	n	n	n	n	n	n	n	n	n	n
hw							n	n	n	**m**	n	n	n	n	n	n	n	n	n	n	n	n	**m**	n	n	n	n	n	n	n	n	n	n	n	n	n
kt								n	n	n	n	n	n	n	n	n	**m**	n	n	n	n	n	**m**	n	n	n	n	n	n	n	n	n	n	n	n	n
fd									n	n	n	**m**	n	n	**m**	n	n	n	n	n	n	n	n	n	n	n	n	n	n	n	n	n	n	n	n	n
sm										n	n	n	n	n	**m**	n	n	n	n	n	n	n	n	n	n	n	n	**m**	n	n	n	n	n	n	n	n
zl											n	n	n	n	**m**	n	n	n	n	n	n	n	n	n	**m**	n	n	n	n	n	n	n	n	n	n	n
kz												n	n	n	n	**m**	n	n	n	n	n	n	n	n	n	n	n	n	n	n	n	n	n	n	n	n
fk													n	n	n	n	n	n	n	n	n	n	n	n	n	n	n	n	n	n	n	n	n	n	n	n
ps														n	n	n	n	n	n	n	n	n	n	n	n	n	n	n	n	n	n	n	n	n	n	n
nb															**m**	**m**	n	**m**	n	n	n	n	n	n	n	n	n	n	n	n	n	n	n	n	n	n
nr																n	n	n	n	n	n	n	n	n	n	n	n	n	n	n	n	n	n	n	n	n
kw																	n	**m**	n	n	n	n	n	n	n	n	n	n	**m**	n	n	n	n	n	n	n
ku																		n	n	n	n	n	n	n	n	n	n	n	n	n	n	n	n	n	n	n
kl																			n	n	n	n	n	n	n	n	n	n	n	n	n	n	n	n	n	n
zm																				n	n	n	n	n	n	n	n	n	n	n	n	n	n	n	n	n
ky																					n	n	n	n	n	n	n	n	**m**	n	n	n	n	n	n	n
rh																						n	n	n	n	n	n	n	n	n	n	n	n	n	n	n
rs																							n	n	n	n	n	n	n	n	n	n	n	n	n	n
ml																								n	n	n	n	n	n	n	n	n	n	n	n	n
jn																									n	n	n	n	n	n	n	n	n	n	n	n
mk																										n	**m**	**m**	n	n	n	n	n	n	n	n
wl																											n	n	n	n	n	n	n	n	n	n
re																												n	n	n	n	n	n	n	n	n
se																													n	n	n	n	n	n	n	n
sb																														**m**	n	n	n	n	n	n
be																															n	n	n	n	n	n
fl																																n	n	n	n	n
bt																																	n	n	n	n
ht																																		n	n	n
jl																																			n	n
vr																																				n
kg																																				

Oct-Dec 2009

bCRI	nk	ms	zf	kt	sq	tk	hw	fd	sm	zl	kz	FK	zg	ps	zd	nb	nr	kw	ku	kl	zm	ky	ka	rh	rs	ml	jn	mk	wl	re	se	sb	be	fl	ht	hl	jl	vr	gl	tj	an
nk		-2.8	17.3	-2	-4.8	45.5	-3.1	-0.9	-6.2	-6.2	-12	-8.2	-6.2	0	0	2.57	-6.4	-6	38.3	1.9	-5.5	0	0	19.6	0	-23	4.39	0	-7.8	0	-33	11.9	0	0	4.25	0	31.1	0	0	0	0
ms			0	0.55	0.9	-16	-2.8	-6	0	0	-3.8	-4.8	0	-3.3	0	7.95	0	0	0	16.8	0	0	0	0	1.31	-8.2	0	5.07	-8.2	3.91	0	0	0	0	0	0	3.93	0	0	0	0
zf				0	1.68	0	-5	0.54	0	0.5	2.21	0.58	0	0	0	0	0	0	0	0	-23	0	0	0	0	0	0	0	0	0	0	0	0	0	0	0	3.36	0	0	0	1.99
kt					0	0	16.1	9.75	-11	-1.5	-3	-2.4	0.49	0	0	0	0.84	0	56.4	0	0	0	0	-8.9	0	0	0	3.76	12.5	5.21	0	11.9	0	0	1.41	0	0	0	0	0	4.52
sq						0	11.5	5.97	0	4.21	1.66	9.49	0	-2.9	0	5.07	-5.8	-14	-19	-7.2	0	0	0	0	0	2.56	0	0	0	8.81	0	0	0	0	0	0	0	0	0	0	0
tk							2.55	-2.9	-13	0	1.11	0	0	0.56	0	0	0	0	0	0	0	0	0	0	0	0	0	0	0	0	0	0	0	0	0	0	0	0	0	0	0
hw								-8.2	-2.1	-1.4	4.33	-1.7	0	-2.9	0	-7.2	18.3	0	1.14	3.81	-7.2	0	0	-7.2	0	-2.3	0	12.4	-17	40.1	0	11.9	15.3	-14	18.8	0	6.79	-14	0	-7.2	-15
fd									3.24	0	0.55	7.86	0	0	0	-25	-19	0	1.38	0	1.38	0	0	0	0	2.56	0	0	0	-16	0	1.38	0	17.7	2.77	0	0	1.38	0	0	4.15
sm										0	3.57	0	0	0	0	0	0	0	0	-12	0	0	0	0	0	0	0	5.8	-54	0	77.8	2.04	0	0	-13	0	-19	0	0	0	0
zl											0.55	8.46	-3.2	0	0.5	0	19.7	1.27	1.27	0	-40	0	0	-8	4.31	-24	-8	10.1	0	0	0	0	0	0	0	0	4.63	0	0	0	0
kz												-7.4	0	0	0	0	0	22.2	0	0	1.41	1.41	0	0	0	-18	0	0	0	-18	0	1.41	0	0	-19	0	4.77	0	0	0	0
FK													-3.7	0	0	0	-9.2	1.47	0	-29	-22	0	0	0	0	1.47	-9.2	5.23	1.47	-17	0	0	0	9.62	0	0	0	0	0	0	-9.2
ps														0	0	-17	2.62	0	0	-6.6	25.2	0	0	0	0	25	0	3.76	0	1.25	0	0	0	0	0	0	0	1.25	0	0	0
zg															0	0	0	0	0	0	0	0	0	0	0	0	0	1.42	0	2.72	0	0	0	0	0	0	0	0	0	0	0
zd																0	0	0	0	0	0	0	0	0	0	0	0	0	0	1.3	0	0	0	0	0	0	0	0	0	0	0
nb																	40.7	-3.4	0	24.1	4.22	0	0	-51	0	5.5	-14	0	-14	2.8	0	-122	0	0	0	0	0	0	0	0	0
nr																		-32	23	-17	0	0	0	0	0	5.5	0	0	0	-14	0	0	0	0	0	0	0	0	0	0	0
kw																			0	-18	0	0	0	0	-18	-31	0	0	-36	0	0	0	0	0	0	0	0	0	0	0	0
ku																				33.5	0	0	0	0	6.53	-31	0	0	0	0	0	0	0	0	0	0	0	0	0	0	0
kl																					0	-4.8	0	0	0	-46	0	0	0	0	4.09	0	0	0	4.09	0	0	0	0	0	0
zm																						0	0	0	0	0	0	0	-20	0	0	0	0	0	0	0	0	0	0	0	0
ky																							10.6	0	0	0	0	0	0	0	0	0	0	0	0	0	0	0	0	0	0
ka																								0	0	0	0	0	0	0	0	0	0	0	0	0	0	0	0	0	0
rh																									6.53	0	0	0	0	0	0	0	0	0	0	0	0	0	0	0	0
rs																										0	0	0	0	0	0	0	0	0	0	0	0	0	0	0	0
ml																											0	0	37.5	5.44	0	85.6	0	0	0	0	0	0	0	0	0
jn																												0	0	0	0	0	0	0	0	0	0	0	0	0	0
mk																													6.98	51.6	0	0	0	0	0	0	0	0	0	0	0
wl																														29.4	6.98	0	32.9	0	0	0	0	0	0	0	0
re																															2.8	0	32.9	0	0	0	0	42.1	0	0	0
se																																0	0	0	0	0	0	0	0	0	0
sb																																	0	0	0	0	0	0	0	0	0
be																																		0	0	0	0	0	0	0	0
fl																																			0	0	0	0	0	0	0
ht																																				0	-34	0	0	0	0
hl																																					0	0	0	0	0
jl																																						0	0	0	0
vr																																							0	0	0
gl																																								0	0
tj																																									0
an																																									

xacty	nk	ms	zf	kt	sq	tk	hw	fd	sm	zl	kz	FK	zg	ps	zd	nb	nr	kw	ku	kl	zm	ky	ka	rh	rs	ml	jn	mk	wl	re	se	sb	be	fl	ht	hl	jl	vr	gl	tj	an
nk		n	**m**	n	n	**m**	n	n	n	n	n	n	n	n	n	**m**	n	n	n	n	n	n	n	**m**	n	n	n	n	n	n	n	n	n	n	**m**	n	**m**	n	n	n	n
ms			n	n	n	n	n	n	n	n	n	n	n	n	n	**m**	n	n	n	n	n	n	n	n	n	n	n	**m**	n	n	n	n	n	n	n	n	n	n	n	n	n
zf				n	**m**	n	n	n	n	n	n	n	n	n	n	n	n	n	n	n	n	n	n	n	n	n	n	n	n	n	n	n	n	n	n	n	n	n	n	n	n
kt					n	n	**m**	**m**	n	n	n	n	n	n	n	n	n	n	**m**	n	n	n	n	n	n	n	n	n	**m**	n	n	n	n	n	n	n	n	n	n	n	**m**
sq						n	n	**m**	n	**m**	n	**m**	n	n	n	**m**	n	n	n	n	n	n	n	n	n	n	n	n	n	**m**	n	n	n	n	n	n	n	n	n	n	n
tk							**m**	n	n	n	n	n	n	n	n	n	n	n	n	n	n	n	n	n	n	n	n	n	n	n	n	n	n	n	n	n	n	n	n	n	n
hw								n	n	n	**m**	n	n	n	n	n	**m**	n	n	n	n	n	n	n	n	n	n	**m**	n	**m**	n	n	n	n	**m**	n	**m**	n	n	n	n
fd									**m**	n	n	**m**	n	n	n	n	n	n	n	n	n	n	n	n	n	n	n	n	n	n	n	n	n	**m**	n	n	n	n	n	n	n
sm										n	**m**	n	n	n	n	n	n	n	n	n	n	n	n	n	n	n	n	**m**	n	n	**m**	n	n	n	n	n	n	n	n	n	n
zl											n	n	n	n	n	n	**m**	n	n	n	n	n	n	n	**m**	**m**	**m**	**m**	n	n	n	n	n	n	n	n	**m**	n	n	n	n
kz												n	n	n	n	n	n	**m**	n	n	n	n	n	n	n	n	n	n	n	n	n	n	n	n	n	n	**m**	n	n	n	n
FK													n	n	n	n	n	n	n	n	n	n	n	n	n	n	n	**m**	n	n	n	n	n	**m**	n	n	n	n	n	n	n
ps														n	n	n	**m**	n	n	n	n	n	n	n	n	n	n	n	n	n	n	n	n	n	n	n	n	n	n	n	n
zg															n	n	n	n	n	n	n	n	n	n	n	n	n	n	n	**m**	n	n	n	n	n	n	n	n	n	n	n
zd																n	n	n	n	n	n	n	n	n	n	n	n	n	n	n	n	n	n	n	n	n	n	n	n	n	n
nb																	**m**	n	n	**m**	n	n	n	n	n	n	n	n	n	n	n	n	n	n	n	n	n	n	n	n	n
nr																		n	n	n	n	n	n	n	n	n	n	n	n	n	n	n	n	n	n	n	n	n	n	n	n
kw																			n	n	n	n	n	n	n	n	n	n	n	n	n	n	n	n	n	n	n	n	n	n	n
ku																				**m**	n	n	n	n	n	n	n	n	n	n	n	n	n	n	n	n	n	n	n	n	n
kl																					n	n	n	n	n	n	n	n	n	n	n	n	n	n	n	n	n	n	n	n	n
zm																						n	n	n	n	n	n	n	n	n	n	n	n	n	n	n	n	n	n	n	n
ky																							n	n	n	n	n	n	n	n	n	n	n	n	n	n	n	n	n	n	n
ka																								n	n	n	n	n	n	n	n	n	n	n	n	n	n	n	n	n	n
rh																									n	n	n	n	n	n	n	n	n	n	n	n	n	n	n	n	n
rs																										n	n	n	n	n	n	n	n	n	n	n	n	n	n	n	n
ml																											n	n	**m**	n	n	n	n	n	n	n	n	n	n	n	n
jn																												n	n	n	n	n	n	n	n	n	n	n	n	n	n
mk																													n	**m**	n	n	n	n	n	n	n	n	n	n	n
wl																														**m**	n	n	n	n	n	n	n	n	n	n	n
re																															n	n	n	n	n	n	n	**m**	n	n	n
se																																n	n	n	n	n	n	n	n	n	n
sb																																	n	n	n	n	n	n	n	n	n
be																																		n	n	n	n	n	n	n	n
fl																																			n	n	n	n	n	n	n
ht																																				n	n	n	n	n	n
hl																																					n	n	n	n	n
jl																																						n	n	n	n
vr																																							n	n	n
gl																																								n	n
tj																																									n
an																																									

Jan - Mar 2010

bCRI	nk	ms	zf	kt	hw	sq	fd	sm	zl	kz	FK	ps	zd	zg	kl	ku	kw	ky	nb	nr	zm	an	fl	jn	ml	mK	re	rh	rs	sb	wl	ht	jl	se	tj	vr
nk		-14	48.1	-1.6	-8.7	-15	-6.7	0	-9.6	-3.3	0.44	-3.3	-3.3	-3.3	23.4	6.73	16.9	6.47	-21	-46	7.35	0	0	0	6.56	0	0	-6.5	-7.6	0	0	0	16.9	0	0	0
ms			3.66	0.45	-6.4	1.23	1.75	7.27	0.87	1.96	-3.3	0.46	0	0	2.21	0	2.34	0	34.2	1.94	4.8	0	0	0	0	0	1.46	0	0	0	0	0	1.6	0	0	0
zf				0.99	0.42	-3.5	0	-3.9	-3.9	0.65	0.89	0	0	0	0	1.67	2.34	0	1.46	0	0	0	0	0	0	0	0	0	0	0	0	0	2.62	0	0	0
kt					6.84	1.4	1.76	0	0.43	0	0.89	0.92	0	7.41	0	24.1	0	0	0	0	-12	0	0	0	7.89	-7.4	17.5	0	0	0	4.56	0	0	0	0	0
hw						14.3	-11	0	13.3	-11	-3.1	6.85	0	0	-7	-7	2.34	-7	-14	4.55	-3.1	0	0	0	1.3	0	9.43	0	0	0	1.3	5.47	3.93	0	0	7.59
sq							4.41	0	-8.9	10.4	0	0	0	0.58	-25	-6.8	0	-35	-12	0	2.06	0	0	0	1.26	0	0	0	0	0	0	-6.8	2.53	0	0	0
fd								1.2	0.87	0	0	0.92	0	0	0	0	0	0	0	0	1.34	0	14.6	0	0	0	0	0	0	0	0	0	6.65	0	0	0
sm									-3.1	0	0	6.43	0	0	0	0	0	0	21.8	0	0	0	0	0	0	0	0	0	0	0	6.9	0	0	68.5	0	0
zl										-1.4	0	-6	0	-3.1	-12	19.8	0	0	-16	-20	0	0	13.2	0	0	0	19.8	0	0	0	0	0	9.24	0	0	0
kz											0	-8.1	0	0	0	0	48.2	0	-7.9	0	0	0	0	0	0	0	-19	0	0	0	0	0	0	0	0	0
FK												-3.2	0	0	1.36	-19	-20	-7.3	-15	-43	0	0	15.9	0	-7.3	0	-5.9	0	0	0	0	0	0	0	0	0
ps													0	0	0	0	0	0	-16	0	0	0	0	-31	0	0	1.42	0	0	0	-24	0	1.42	-28	0	0
zd														0	0	0	0	0	0	0	0	0	0	0	0	0	0	0	0	0	0	0	0	0	0	0
zg															0	0	0	0	0	0	28.1	0	0	0	0	0	0	0	0	0	0	0	0	0	0	0
kl																6.15	25.3	0	2.63	-36	6.15	0	0	0	0	0	0	-75	-36	0	0	0	0	0	0	0
ku																	0	0	4.08	0	-1.2	0	0	0	0	0	0	0	0	0	0	0	0	0	0	0
kw																		0	36.7	5.41	0	0	0	0	0	0	-38	0	0	0	0	0	0	0	0	0
ky																			0	0	0	0	0	0	0	0	0	0	0	0	0	0	0	0	0	0
nb																				0	0	0	0	0	0	0	4.06	0	23	0	0	0	0	0	0	0
nr																					0	-32	0	0	-79	0	-32	0	0	0	0	0	0	0	0	0
zm																						0	0	0	0	0	0	-75	0	0	0	0	0	0	0	0
an																							0	0	0	0	0	0	0	0	0	0	0	0	0	0
fl																								0	102	0	0	0	0	0	0	0	0	0	0	0
jn																									0	0	0	0	0	0	0	0	0	0	0	0
ml																										0	0	14.3	0	0	0	0	14.3	0	0	0
mk																											0	0	0	0	0	0	0	0	0	0
re																												0	0	0	88.2	0	0	0	0	0
rh																													72.2	0	0	0	0	0	0	0
rs																														0	0	0	0	0	0	0
sb																															0	0	0	0	0	0
wl																																0	0	0	0	0
ht																																	0	0	0	0
jl																																		0	0	0
se																																			0	0
tj																																				0
vr																																				

xacty	nk	ms	zf	kt	hw	sq	fd	sm	zl	kz	FK	ps	zd	zg	kl	ku	kw	ky	nb	nr	zm	an	fl	jn	ml	mK	re	rh	rs	sb	wl	ht	jl	se	tj	vr
nk		n	**m**	n	n	n	n	n	n	n	n	n	n	n	**m**	n	**m**	n	n	n	n	n	n	n	**m**	n	n	n	n	n	n	n	**m**	n	n	n
ms			**m**	n	n	n	n	n	n	n	n	n	n	n	n	n	n	n	**m**	n	n	n	n	n	n	n	n	n	n	n	n	n	n	n	n	n
zf				**m**	n	n	n	n	n	n	n	n	n	n	n	n	n	n	n	n	n	n	n	n	n	n	n	n	n	n	n	n	n	n	n	n
kt					**m**	n	**m**	n	n	n	n	n	n	**m**	n	**m**	n	n	n	n	n	n	n	n	**m**	n	n	n	n	n	n	n	n	n	n	n
hw						**m**	n	n	**m**	n	n	**m**	n	n	n	n	n	n	n	**m**	n	n	n	n	n	n	**m**	n	n	n	n	**m**	**m**	n	n	**m**
sq							n	n	n	n	n	n	n	n	n	n	n	n	n	n	n	n	n	n	n	n	n	n	n	n	n	n	n	n	n	n
fd								**m**	**m**	n	n	n	n	n	n	n	n	n	n	n	n	n	**m**	n	n	n	n	n	n	n	n	n	**m**	n	n	n
sm									n	n	n	n	n	n	n	n	n	n	n	n	n	n	n	n	n	n	n	n	n	n	**m**	n	n	**m**	n	n
zl										n	n	n	n	n	n	n	n	n	n	n	n	n	n	n	n	n	n	n	n	n	n	n	**m**	n	n	n
kz											n	n	n	n	n	n	**m**	n	n	n	n	n	n	n	n	n	n	n	n	n	n	n	n	n	n	n
FK												n	n	n	n	n	n	n	n	n	n	n	**m**	n	n	n	n	n	n	n	n	n	n	n	n	n
ps													n	n	n	n	n	n	n	n	n	n	n	n	n	n	n	n	n	n	n	n	n	n	n	n
zd														n	n	n	n	n	n	n	n	n	n	n	n	n	n	n	n	n	n	n	n	n	n	n
zg															n	n	n	n	n	n	**m**	n	n	n	n	n	n	n	n	n	n	n	n	n	n	n
kl																n	**m**	n	n	n	n	n	n	n	n	n	n	n	n	n	n	n	n	n	n	n
ku																	n	n	n	n	n	n	n	n	n	n	n	n	n	n	n	n	n	n	n	n
kw																		n	n	n	n	n	n	n	n	n	n	n	n	n	n	n	n	n	n	n
ky																			n	n	n	n	n	n	n	n	n	n	n	n	n	n	n	n	n	n
nb																				n	n	n	n	n	n	n	n	n	n	n	n	n	n	n	n	n
nr																					n	n	n	n	n	n	n	n	n	n	n	n	n	n	n	n
zm																						n	n	n	n	n	n	n	n	n	n	n	n	n	n	n
an																							n	n	n	n	n	n	n	n	n	n	n	n	n	n
fl																								n	**m**	n	n	n	n	n	n	n	n	n	n	n
jn																									n	n	n	n	n	n	n	n	n	n	n	n
ml																										n	n	n	n	n	n	n	n	n	n	n
mk																											n	n	n	n	n	n	n	n	n	n
re																												n	n	n	**m**	n	n	n	n	n
rh																													n	n	n	n	n	n	n	n
rs																														n	n	n	n	n	n	n
sb																															n	n	n	n	n	n
wl																																n	n	n	n	n
ht																																	n	n	n	n
jl																																		n	n	n
se																																			n	n
tj																																				n
vr																																				

Apr - Jun 2010

bCRI	nk	ms	zf	kt	sq	hw	fd	sm	zl	kz	FK	zg	ps	zd	nb	nr	kw	ku	kl	zm	ky	rh	rs	ml	jn	mk	wl	re	se	sb	be	fl	ht	jl	vr	gl	tj	an	ok	dg
nk		11.6	8.08	-1	-0	3.05	-4.5	0	-5.8	-4.5	-4.9	-3.3	0	0	79.7	-7.5	0.06	3.18	4.49	7.95	0	0	0	0	4.69	0	-15	-14	0	0	0	0	0	-3.7	0	0	0	0	0	-7.5
ms			-6.6	-0.9	-11	-4.7	-1.5	0	3.17	1.3	-4.9	0.38	0	0	25.9	0	3.09	0	0	22.6	0	0	0	0	0	0	0	0	0	0	0	0	0	0	0	0	0	0	-9.5	0
zf				-4.5	-3	16.2	0.83	0	0	0	-4.1	0	-4.5	0	0	0	0	0	0	-14	0	0	0	0	0	0	0	0	0	0	0	0	0	-100	0	0	0	0	0	0
kt					0.34	-4.9	-0.8	0.86	0.41	-1.2	-1.2	0	-3.3	0	6.37	0	0	0	-7.5	26.9	0	0	0	0	4.69	0	0	6.84	0	0	0	0	0	20.8	0	0	0	0	11.1	1.18
sq						11.7	5.87	0	-2.8	2.2	4.36	0.38	3.5	0	15.1	0	0	0	0	-14	21.8	0	0	0	0	0	0	21.8	0	0	89.8	0	0	-8	0	0	0	0	0	0
hw							0.8	0	-1.5	-0.3	-1.6	0	3.28	0	0	0	22	0	1.65	2.35	0	0	0	3.19	0	0	0	42.6	-6.3	0	0	0	20.4	14.5	0	0	0	0	-15	-7.5
fd								0	0	-0.9	2.56	-1.4	-1.4	0.42	-7.9	0	0	0	1.29	-5.6	-17	0	0	0	0	0	0	1.29	0	0	0	0	0	1.29	0	0	0	0	0	-8.2
sm									8.96	1.29	0	0	0	0	-17	0	0	0	-17	0	-17	0	0	0	0	0	0	0	0	0	0	0	-17	0	0	0	0	0	-17	0
zl										1.43	-1.5	-1.1	0.38	2.83	-52	0	0	0	-16	-15	-24	0	0	-3.8	0	0	-17	1.71	0	0	0	0	0	0	0	0	0	0	5.21	28.3
kz											-1.9	0	0	-1.1	-16	0	18.7	0	0	1.34	0	0	0	0	0	0	0	0	0	0	0	0	0	0	0	0	0	0	0	1.34
FK												0	3.73	-3.7	0	0	1.33	0	-8.5	-8.5	-8.5	0	0	0	0	0	0	1.33	0	0	0	19.7	0	0	0	0	0	0	0	-8.5
ps													0	0.38	-7.9	0	0	0	0	0	0	0	0	-7.4	0	0	0	1.71	0	0	0	0	0	0	0	0	0	0	0	0
zg														0.35	13.7	0	0	0	0	0	0	0	0	0	0	0	0	-9.7	0	0	0	0	0	0	0	0	0	0	0	0
zd															0	0	-10	0	1.09	0	0	0	1.09	0	0	0	-19	0	0	0	0	0	0	0	0	0	0	0	0	0
nb																68.2	98.1	0	54.7	4.78	82.5	0	0	0	0	-25	5.42	3.18	0	0	0	0	0	0	0	0	0	0	-51	0
nr																	0	0	0	0	0	0	0	0	0	0	0	3.18	0	0	0	0	0	0	0	0	0	0	0	0
kw																		8.86	65	0	-83	0	0	-41	0	0	0	2.96	0	0	0	0	0	0	0	0	0	0	-65	0
ku																			0	0	0	0	0	0	0	0	0	0	0	0	0	0	0	0	0	0	0	0	0	0
kl																				0	0	0	0	0	8.71	0	0	0	0	0	0	0	0	0	0	0	0	0	0	0
zm																					37.8	0	0	0	0	0	0	0	0	0	0	0	0	0	0	0	0	0	0	0
ky																						0	0	0	0	0	0	0	0	21.1	0	0	5.07	0	0	0	0	0	-56	0
rh																							0	0	0	0	0	0	0	0	0	0	0	0	0	0	0	0	0	0
rs																								0	0	0	0	0	0	0	0	0	0	0	0	0	0	0	0	0
ml																									0	0	18.3	6.91	0	0	0	0	0	0	0	0	0	0	0	0
jn																										0	0	0	0	0	0	0	0	0	0	0	0	0	0	0
mk																											0	13.5	0	0	0	0	0	0	0	0	0	0	0	0
wl																												3.18	0	0	0	0	0	0	0	0	0	0	67.1	0
re																													0	0	0	0	0	0	0	0	0	0	-35	0
se																														0	0	0	0	0	0	0	0	0	-91	0
sb																															0	0	0	0	0	0	0	0	0	0
be																																0	0	0	0	0	0	0	0	0
fl																																	0	0	0	0	0	0	0	0
ht																																		0	0	0	0	0	0	0
jl																																			0	0	0	0	0	0
vr																																				0	0	0	-174	0
gl																																					0	0	0	0
tj																																						0	0	0
an																																							0	0
ok																																								0
dg																																								

bCRI	nk	ms	zf	kt	sq	hw	fd	sm	zl	kz	FK	zg	ps	zd	nb	nr	kw	ku	kl	zm	ky	rh	rs	ml	jn	mk	wl	re	se	sb	be	fl	ht	jl	vr	gl	tj	an	ok	dg
nk		**m**	n	n	n	n	n	n	n	n	n	n	n	n	**m**	n	n	n	**m**	**m**	n	n	n	n	n	n	n	n	n	n	n	n	n	n	n	n	n	n	n	n
ms			n	n	n	n	n	n	**m**	n	n	n	n	n	**m**	n	**m**	n	n	n	n	n	n	n	n	n	n	n	n	n	n	n	n	n	n	n	n	n	n	n
zf				n	n	**m**	n	n	n	n	n	n	n	n	n	n	n	n	n	n	n	n	n	n	n	n	n	n	n	n	n	n	n	n	n	n	n	n	n	n
kt					n	n	n	n	n	n	n	n	n	n	**m**	n	n	n	n	**m**	n	n	n	n	n	n	n	n	n	n	n	n	n	**m**	n	n	n	n	n	n
sq						**m**	**m**	n	n	**m**	**m**	n	n	n	n	n	n	n	n	n	n	n	n	n	n	n	n	n	n	n	**m**	n	n	n	n	n	n	n	n	n
hw							**m**	n	n	n	n	n	n	n	n	n	**m**	n	n	n	n	n	n	**m**	n	n	n	**m**	n	n	n	n	n	n	n	n	n	n	n	n
fd								n	n	n	**m**	n	n	n	n	n	n	n	n	n	n	n	n	n	n	n	n	n	n	n	n	n	n	n	n	n	n	n	n	n
sm									**m**	**m**	n	n	n	n	n	n	n	n	n	n	n	n	n	n	n	n	n	n	n	n	n	n	n	n	n	n	n	n	n	n
zl										n	n	n	n	**m**	n	n	n	n	n	n	n	n	n	n	n	n	n	n	n	n	n	n	n	n	n	n	n	n	n	n
kz											n	n	n	n	n	n	**m**	n	n	n	n	n	n	n	n	n	n	n	n	n	n	n	n	n	n	n	n	n	n	n
FK												n	n	n	n	n	n	n	n	n	n	n	n	n	n	n	n	n	n	n	n	**m**	n	n	n	n	n	n	n	n
ps													n	n	n	n	n	n	n	n	n	n	n	n	n	n	n	n	n	n	n	n	n	n	n	n	n	n	n	n
zg														n	n	n	n	n	n	n	n	n	n	n	n	n	n	**m**	n	n	n	n	n	n	n	n	n	n	n	n
zd															n	n	n	n	n	n	n	n	n	n	n	n	n	n	n	n	n	n	n	n	n	n	n	n	n	n
nb																**m**	**m**	n	**m**	**m**	n	n	n	n	n	n	n	n	n	n	n	n	n	n	n	n	n	n	n	n
nr																	n	n	n	n	n	n	n	n	n	n	n	n	n	n	n	n	n	n	n	n	n	n	n	n
kw																		**m**	**m**	n	n	n	n	n	n	n	n	n	n	n	n	n	n	n	n	n	n	n	n	n
ku																			n	n	n	n	n	n	n	n	n	n	n	n	n	n	n	n	n	n	n	n	n	n
kl																				n	n	n	n	n	n	n	n	n	n	n	n	n	n	n	n	n	n	n	n	n
zm																					n	n	n	n	n	n	n	n	n	n	n	n	n	n	n	n	n	n	n	n
ky																						n	n	n	n	n	n	n	n	**m**	n	n	n	n	n	n	n	n	n	n
rh																							n	n	n	n	n	n	n	n	n	n	n	n	n	n	n	n	n	n
rs																								n	n	n	n	n	n	n	n	n	n	n	n	n	n	n	n	n
ml																									n	n	**m**	**m**	n	n	n	n	n	n	n	n	n	n	n	n
jn																										n	n	n	n	n	n	n	n	n	n	n	n	n	n	n
mk																											n	**m**	n	n	n	n	n	n	n	n	n	n	n	n
wl																												n	n	n	n	n	n	n	n	n	n	n	**m**	n
re																													n	n	n	n	n	n	n	n	n	n	n	n
se																														n	n	n	n	n	n	n	n	n	n	n
sb																															n	n	n	n	n	n	n	n	n	n
be																																n	n	n	n	n	n	n	n	n
fl																																	n	n	n	n	n	n	n	n
ht																																		n	n	n	n	n	n	n
jl																																			n	n	n	n	n	n
vr																																				n	n	n	n	n
gl																																					n	n	n	n
tj																																						n	n	n
an																																							n	n
ok																																								n
dg																																								
